# Metabolic imaging using hyperpolarized ^13^C‐pyruvate to assess sensitivity to the B‐Raf inhibitor vemurafenib in melanoma cells and xenografts

**DOI:** 10.1111/jcmm.14890

**Published:** 2019-12-13

**Authors:** Stefania Acciardo, Lionel Mignion, Estelle Lacomblez, Céline Schoonjans, Nicolas Joudiou, Florian Gourgue, Caroline Bouzin, Jean‐François Baurain, Bernard Gallez, Bénédicte F. Jordan

**Affiliations:** ^1^ Biomedical Magnetic Resonance (REMA) Group Louvain Drug Research Institute (LDRI) Université catholique de Louvain (UCLouvain) Brussels Belgium; ^2^ Nuclear and Electron Spin Technologies (NEST) Platform Louvain Drug Research Institute (LDRI) Université catholique de Louvain (UCLouvain) Brussels Belgium; ^3^ Imaging platform 2IP Institut de Recherche Expérimentale et Clinique (IREC) Université catholique de Louvain (UCLouvain) Brussels Belgium; ^4^ Molecular Imaging and Radiation Oncology (MIRO) Group Institute de Recherche Expérimentale et Clinique (IREC) Université Catholique de Louvain (UCLouvain) Brussels Belgium

**Keywords:** BRAF inhibition, hyperpolarized ^13^C‐pyruvate, melanoma, resistance, tumour metabolism

## Abstract

Nearly all melanoma patients with a BRAF‐activating mutation will develop resistance after an initial clinical benefit from BRAF inhibition (BRAFi). The aim of this work is to evaluate whether metabolic imaging using hyperpolarized (HP) ^13^C pyruvate can serve as a metabolic marker of early response to BRAFi in melanoma, by exploiting the metabolic effects of BRAFi. Mice bearing human melanoma xenografts were treated with the BRAFi vemurafenib or vehicle. In vivo HP ^13^C magnetic resonance spectroscopy was performed at baseline and 24 hours after treatment to evaluate changes in pyruvate‐to‐lactate conversion. Oxygen partial pressure was measured via electron paramagnetic resonance oximetry. Ex vivo qRT‐PCR, immunohistochemistry and WB analysis were performed on tumour samples collected at the same time‐points selected for in vivo experiments. Similar approaches were applied to evaluate the effect of BRAFi on sensitive and resistant melanoma cells in vitro, excluding the role of tumour microenvironment. BRAF inhibition induced a significant increase in the HP pyruvate‐to‐lactate conversion in vivo, followed by a reduction of hypoxia. Conversely, the conversion was inhibited in vitro, which was consistent with BRAFi‐mediated impairment of glycolysis. The paradoxical increase of pyruvate‐to‐lactate conversion in vivo suggests that such conversion is highly influenced by the tumour microenvironment.

## 1 INTRODUCTION

Melanoma is the most aggressive form of skin cancer, with increasing incidence rates and poor prognosis in the presence of metastasis.^1^ More than half of melanomas harbour a mutation in the BRAF gene, which consists in most cases in a substitution of valine to glutamic acid in position 600 (V600E mutation).^2^ Two main treatment options are available for advanced melanoma patients harbouring a BRAF mutation: targeted therapies, consisting of BRAFi and MEKi, and immunotherapies. Immunotherapies have displayed long‐term effect on a subset of patients but, to date, there are no tools to identify those patients that will benefit from immunotherapies. On the other hand, targeted therapies have immediate effect in term of tumour shrinkage, but the benefit is short‐term as resistance occurs after few months in almost every case. As a further matter, some patients do not respond at all to BRAF inhibition because of intrinsic resistance to the treatment. The addition of a MEK inhibitor to the regimen was initially identified as a promising approach to overcome BRAFi‐resistance, and hence the combination of BRAFi/MEKi has been approved.^4^, ^5^, ^6^ However, in numerous cases the BRAFi/MEKi combination just delays the emergence of resistance as observed when we compare the median progression‐free survival of patients receiving the monotherapy (7.3 months) vs the combination (14.9 months).^3^

More recently, re‐challenging non‐responding tumour after a drug holiday period has been suggested as an alternative therapeutic strategy in BRAF‐mutated melanomas^8^, ^9^ and it has been shown to be beneficial in a subset of patients.^10^, ^11^, ^12^, ^13^, ^14^, ^15^ However, safe longitudinal biomarkers to identify the patients who may benefit from the intermittent treatment, or to establish the optimal drug holiday duration, are missing.

Several studies have recently helped deciphering the interplay between oncogenic MAPK signalling, melanoma metabolism and BRAFi‐resistance. Apart from a small subset of melanomas, BRAF mutations are associated with increased glycolysis and attenuated oxidative phosphorylation, and such balance is reversed upon treatment with BRAFi.^16^, ^17^, ^18^, ^19^, ^20^ Resistance to BRAFi seems to be accompanied by precise metabolic changes as well: higher reliance on oxidative phosphorylation,^21^, ^22^, ^23^, ^24^ glutamine dependency^21^, ^25^, ^26^ and up‐regulated serine metabolism.^26^, ^27^

Besides suggesting novel targetable metabolic vulnerabilities, the fast‐increasing knowledge in tumour metabolism will hopefully help identifying candidate biomarkers of clinical utility. To date, the lack of validated markers exploiting the aforementioned metabolic alterations to discriminate responding and non‐responding melanomas in patients still hinders the long‐term effectiveness of current treatment options and represents a barrier to the advancement of personalized medicine.

Metabolic markers such as ^13^C magnetic resonance spectroscopy of hyperpolarized substrate may bridge this gap, as they allow to assess crucial metabolic fluxes whose alteration is indicative of treatment response or tumour progression. Notably, hyperpolarization of ^13^C‐enriched metabolites increases ^13^C magnetic resonance spectroscopy (MR) sensitivity by a factor of 10 000, allowing in vivo real‐time assessment of metabolic fluxes. In particular, [1‐^13^C] pyruvate is reduced to [1‐^13^C] lactate via the enzyme lactate dehydrogenase (LDH). This process results in an altered chemical shift that HP MRI can image at uniquely high‐temporal resolution. Hyperpolarized [1‐^13^C] pyruvate has been safely administered in patients, and its conversion into [1‐^13^C] lactate was higher in prostate tumours compared with healthy tissue, which was in agreement with previous preclinical studies.^28^ The use of hyperpolarized [1‐^13^C] pyruvate for metabolic imaging of prostate, breast, brain and cervical cancer and other diseases is currently being evaluated in several clinical trials.^29^ In addition to pyruvate, several other ^13^C enriched substrates have been hyperpolarized, with their conversion being successfully observed in vivo.^29^

The aim of this study is to evaluate whether magnetic resonance spectroscopy of hyperpolarized [1‐^13^C] pyruvate can discriminate between responding and resistant BRAF^V600E^ melanoma cells and xenografts, by exploiting the effect of BRAFi on tumour metabolism.

## 2 METHODS

### 2.1 Cell culture

Sensitive (A375) and resistant (A375R) melanoma cells were treated for 24 hours with 2 µmol/L of the BRAFi vemurafenib or DMSO. The resistant cell line was generated via exposure to increasing concentration of the drug.^30^ Cells were cultured at 37°C in a humidified atmosphere with 5% CO_2_ and maintained in Dulbecco's modified Eagle's medium (DMEM, Thermo Fisher Scientific) supplemented with 10% heat‐inactivated FBS (Thermo Fisher Scientific).

### 2.2 Animal studies

Experiments involving animals were undertaken in accordance with the Belgian law concerning the protection and welfare of the animals and were approved by the UCLouvain ethical committee (agreement reference: UCL/2014/MD/026). All investigators performing in vivo studies successfully completed FELASA C training.

### 2.3 Tumour xenografts

2 × 10^6^ A375 cells in 100 μL of PBS were subcutaneously injected in the right hind paw of 6‐week‐old female nude NMRI mice (Janvier). During inoculation, mice were kept under inhalational anaesthesia with 2.5% isoflurane in 2 L/min airflow. Mice were treated with daily intraperitoneal injection of vemurafenib (50 mg/kg, Active Biochem) or vehicle (DMSO, Sigma‐Aldrich).

### 2.4 Hyperpolarized ^13^C‐MRS

For hyperpolarization experiments, 10 or 40 µL of [1‐^13^C] pyruvic acid (Sigma‐Aldrich) solution containing 15 mmol/L of trityl radical OX63 and 2 mmol/L gadolinium were hyperpolarized by an Oxford Dynamic Nuclear Polarizer (HyperSense) for about an hour at 1.4 K and 3.35 T. The polarized solution was rapidly dissolved in 3 mL of a heated buffer containing 100 mg/l EDTA, 40 mmol/L HEPES, 30 mmol/L NaCl, 80 mmol/L NaOH, 30 mmol/L of non‐HP, unlabelled lactate. 10 µL of the starting pyruvic acid solution was used for in vitro experiments and 40 µL for in vivo studies.

^13^C spectra acquisition and infusion of HP [1‐^13^C] pyruvate (into the cell suspension or in the animal tail vein) were started simultaneously. Spectra were acquired at 37°C every 3 seconds for 210 seconds. ^13^C label exchange between HP [1‐^13^C] pyruvate and [1‐^13^C] lactate was measured as the ratio between the corresponding areas under the curve (AUC), via a homebuilt Matlab routine (The MathWorks Inc).

Following HP experiments, melanoma xenografts were collected and processed for further analysis (immunohistochemistry, WB, qRT‐PCR).

### 2.5 ^13^C‐MRS

For ^13^C‐glucose flux experiments, cells were incubated in DMEM containing 2 mmol/L glutamine, 10 mmol/L of [U‐^13^C]glucose and either the BRAFi vemurafenib (2 µmol/L) or DMSO. Cell growth media was collected after 24 hours and intracellular metabolites were extracted with a methanol:chloroform:water extraction method. ^13^C spectra of the aqueous fraction of cell extracts and cell growth medium were acquired using a Bruker Ascend 600 MHz NMR system equipped with a broadband cryoprobe. The acquisition time was 0.8 seconds with 1024 repetitions and 10 seconds of interpulse delay (1D sequence with inverse gated decoupling using 30° flip angle). Data were processed using MestRenova (Mestrelab Research SL): metabolites were quantified by peak integration relative to internal standards and corrected for protein amount per sample.

### 2.6 In vitro EPR spectroscopy

The effect of BRAFi on the oxygen consumption rate was evaluated on A375 and A375R cells via an X‐band EPR spectrometer operating at 9 GHz (Bruker EMXplus). Cells were incubated with 2 µmol/L of BRAFi for 24 hours; after trypsinization, cells were resuspended in cell growth medium at a concentration of 9 × 10^6^ cells/mL and 100 µL of cell suspension was mixed with 100 µL of 20% w/v of dextran in 0.9% NaCl solution and 8 µL of 0.2 mmol/L ^15^N‐PDT, which served as oxygen sensor. A 75 µL‐glass capillary tube (Hirschmann Labogeräte) was filled with the cell suspension, sealed and inserted into a quartz tube, then placed into the EPR cavity. The cavity was continuously flushed with a gas mixture (400 L/h) at 37°C throughout the spectra acquisition. EPR spectra were acquired every 60 seconds, and pO_2_ values were obtained by measuring the peak‐to‐peak EPR signal linewidths, which was then converted into pO_2_ by means of a calibration curve. Oxygen consumption rate (OCR) was then calculated as the slope of pO_2_ over time curve.

### 2.7 In vivo EPR spectroscopy

For in vivo EPR experiments, when the shortest tumour diameter reached 5 mm, 50 µL of charcoal suspension (100 mg/mL in 0.9% NaCl containing 3% Arabic gum) was injected intratumorally. The day after charcoal injection, mice were randomized into two groups (BRAFi‐treated or control) and longitudinal EPR measurements were started. Spectra were acquired on a 1.15‐GHz EPR spectrometer (ClinEPR). Typical acquisition parameters were as follows: modulation of amplitude 0.4 G, modulation of frequency 21 kHz. During EPR experiments, animals were kept under inhalational anaesthesia with isoflurane (2.5% during anaesthesia induction and 1.2% during maintenance) in 2 L/min airflow. Acquisition was started 5 minutes after setting isoflurane to 1.2%. Tumour pO_2_ values were obtained by measuring the peak‐to‐peak EPR signal linewidth, which was then converted into pO_2_ by means of a calibration curve.

### 2.8 qRT‐PCR

Total RNA was extracted from cells and tumour tissue powder using the TriPure Isolation Reagent (Roche) according to the manufacturer's instructions. The concentration and quality were determined using a NanoDrop Spectrophotometer. 1 μg of total mRNA was reverse‐transcribed into cDNA using the GoScript Reverse Transcriptase System (Promega). Quantitative real‐time PCR was performed using a StepOnePlus Real‐Time PCR System (Thermo Fisher Scientific) using Mastermix Plus for SYBR Assay (Eurogentec). Data were analysed using the 2^−ΔΔCT^ method. Glyceraldehyde 3‐phosphate dehydrogenase (GAPDH) was used as housekeeping gene. Primers for GLUT1, HK2, MCT1, MCT4, LDHA, ALT2, PDK1, MPC1 and c‐MYC are listed in Appendix S1.

### 2.9 Western blot

Adherent A375 and A375R cells were lysed in RIPA buffer (Thermo Scientific) supplemented with 1% protease and phosphatase inhibitors (Thermo Scientific). Protein amount in whole cell lysates was measured with a Pierce™ BCA Protein Assay Kit (Thermo Scientific). Equal amounts of protein were loaded onto 4%‐15% Mini‐PROTEAN^®^ TGX™ Precast Gels (Bio‐Rad). Following electrophoresis in 1× Tris/glycine/SDS running buffer (Bio‐Rad), proteins were transferred to PVDF membranes using the Trans‐Blot^®^ Turbo™ RTA Mini PVDF Transfer Kit (Bio‐Rad) according to the vendor's instructions. Non‐specific binding was blocked by soaking the membranes in 5% BSA in tTBS (1× Tris‐Buffered Saline, 0.1% Tween 20, Bio‐Rad) at room temperature for 1 hour.

Membranes were incubated with primary anti‐HSP90, anti‐LDHA, anti–β‐actin, anti‐HSP90, anti–c‐MYC, (Cell Signaling), anti‐MCT1, anti‐MCT4, anti‐GLUT1 (Abcam) antibodies in tTBS‐BSA 5% at 4°C overnight, followed by incubation with anti‐rabbit or anti‐mouse secondary antibodies (Jackson IR) in tTBS‐BSA 1% at room temperature for 1 hour. Detection was performed using the SuperSignal™ West Pico Plus kit (Thermo Scientific) and an ImageQuant LAS 500 camera (GE Healthcare). Quantification was performed on ImageJ by measuring the integral of the optical density profile of the band of the expected molecular weight. No background correction was performed.

### 2.10 Immunohistochemistry

Melanoma xenografts were fixed in 4% formaldehyde and embedded in paraffin. After rehydration, 5 µm sections were submitted to antigen retrieval using citrate buffer. Sections were then incubated in BSA 5% in TBS/Triton 0,05% to block non‐specific binding, then overnight at 4°C with primary antibodies for CD31 (Cell Signaling Technology). Envision anti‐rabbit secondary polymer antibody was used (Dako). Stained slides were then digitalized using a SCN400 slide scanner (Leica Biosystems) at 20× magnification and analysed using TissueIA software (Leica Biosystems). The quantification algorithm was run in the non‐necrotic part of the tissues.

### 2.11 Statistical analysis

Unpaired *t* tests and two‐way ANOVA analysis followed by Sidak or Dunnett multiple comparisons test were performed via Graphpad Prism 7 (GraphPad Software), with *P* ≤ .05 considered significant. Data normality and variance homogeneity were verified both informally (boxplot and histogram of sample data) and formally via Shapiro‐Wilk test in RStudio (RStudio Inc, v. 1.1.456). Non‐normal data were log‐transformed prior PCA in RStudio. Results are represented as mean ± SEM.

## 3 RESULTS

### 3.1 Hyperpolarized ^13^C MRS detects metabolic changes induced by BRAFi in vivo

**Figure 1:**
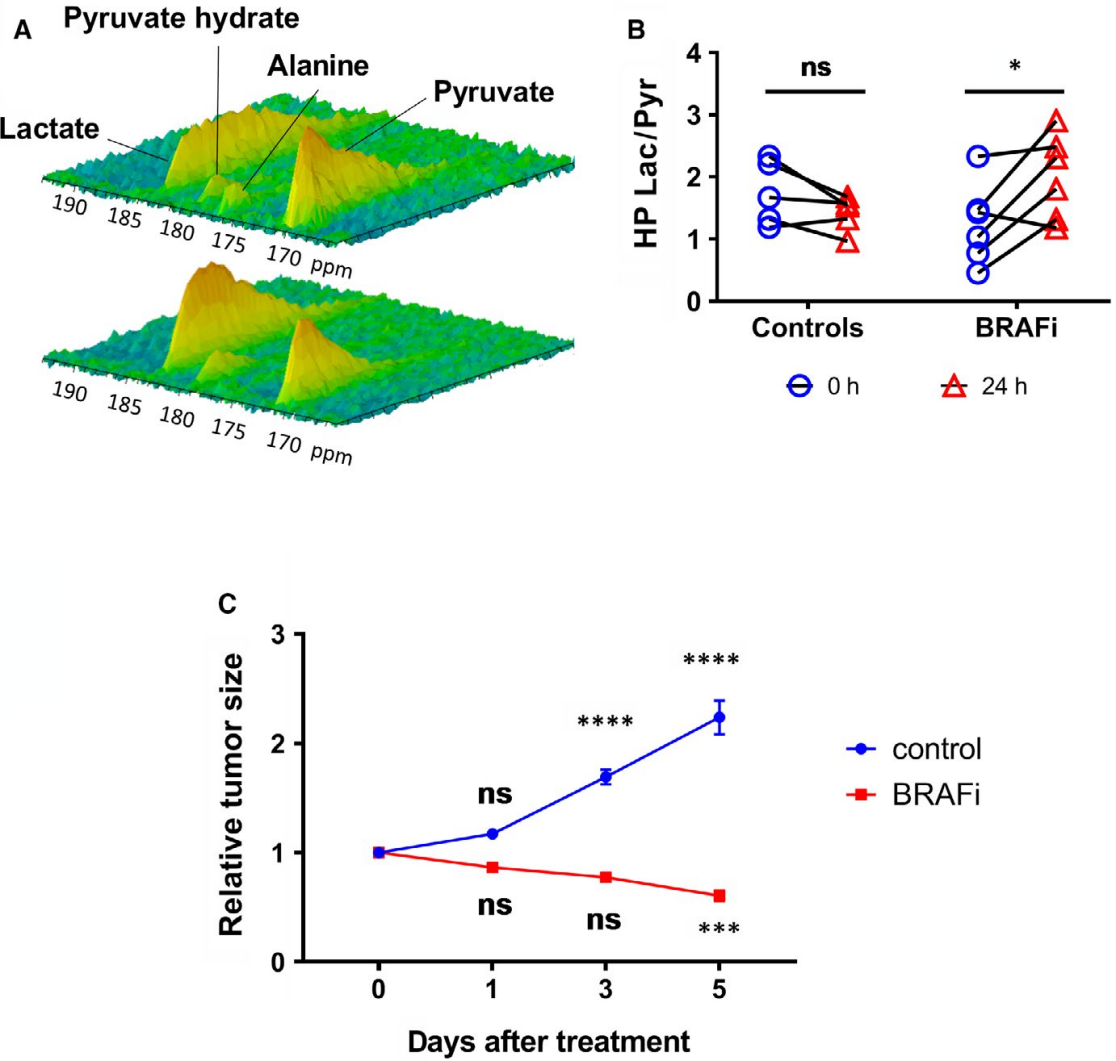
HP pyruvate is an early marker of response to BRAF inhibition. A, Representative spectra of the ^13^C signal time course, obtained from a mouse at baseline (top) and 24 h after a single injection of vemurafenib (bottom). B, ^13^C label exchange between HP pyruvate and lactate (measured as the ratio AUC of [1‐ C] lactate/AUC of [1‐ C] pyruvate) in melanoma xenografts prior treatment and 24 h after injection of the BRAFi vemurafenib (50 mg/kg) or DMSO (two‐way ANOVA, Sidak multiple comparisons test, *P < .05, ns: non‐significant) (n = 6). C, Tumour growth curves obtained from BRAFi‐sensitive melanoma xenografts treated with daily intraperitoneal injection of vemurafenib (50 mg/kg) or DMSO (two‐way ANOVA, Dunnett's multiple comparisons test, **P < .01, **P < .0001) (n = 7). Lac, lactate; pyr, pyruvate

Following intravenous injection of hyperpolarized (HP) [1‐^13^C] pyruvate in mice bearing A375 xenografts, we observed ^13^C signal from [1‐^13^C] pyruvate hydrate, [1‐^13^C] lactate and [1‐^13^C] alanine (Figure 1A). ^13^C signal arising from alanine was only observed in four mice (33%). The ^13^C label exchange between HP pyruvate and lactate increased 24 hours after treatment with vemurafenib (*P* = .3839 in controls and *P* = .0171 in BRAFi‐treated mice) (Figure 1B). This effect occurred before any significant change in tumour volume. The tumour size of control melanoma xenografts became significantly larger 3 days after the start of the experiment (*P* = .3892 at day 1, *P* < .0001 at day 3 and 5), whereas the growth of treated melanoma xenografts was initially stabilized by the treatment, and significant tumour shrinkage occurred after 5 days (*P* = .4395 at day 1, *P* = .0567 at day 3 and *P* = .0005 at day 5) (Figure 1C). To explain the increase in the HP lactate/pyruvate ratio, we have measured protein and mRNA levels of key glycolytic enzymes and transporters in tumour xenografts collected right after the hyperpolarization experiments. Of note, the HP pyruvate‐to‐lactate conversion was increased in  all but one animals after treatment, compared with baseline, but the two groups did not significantly differ at 24 hours (*P* = .3078 in controls versus treated mice at baseline and *P* = .2050 at 24 hours). This highlights the importance of longitudinal, individual monitoring compared with the measurement of averages in groups. This approach, however, is not always feasible when quantifying proteins or mRNA levels in tissues. Therefore, for the ex vivo analysis, we have compared the two groups at 24 hours after treatment with BRAFi or vehicle.

**Figure 2:**
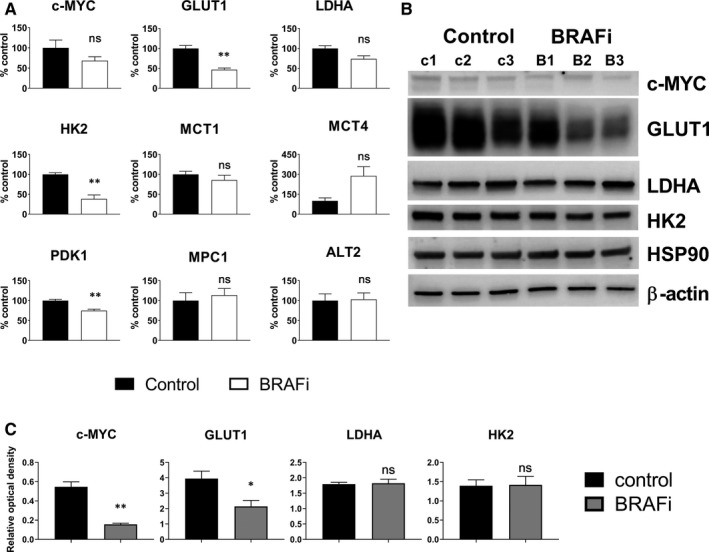
Molecular markers of response to BRAFi ex vivo. A, mRNA levels of glycolysis‐related genes, evaluated in melanoma xenografts collected 24 h after a single injection of the BRAFi vemurafenib (50 mg/kg) (unpaired t test, *P < .05. **P < .01, ns: non‐significant) (n = 3). B, Western blot and C, optical density analysis of c‐MYC, GLUT1, HK2 and LDHA. HSP90 and β‐actin were used as loading control (unpaired t test, *P < .05. **P < .01, ns: non‐significant)

In treated xenografts, the glucose transporter GLUT1 was significantly lower both at the mRNA (*P* = .0010) and protein (*P* = .0440) level, when compared to control xenografts (Figure 2A‐C). Treated xenografts also showed lower mRNA levels of HK2, PDK1 (*P* = .0037 and *P* = .0046, respectively) and a significant reduction in c‐MYC protein levels (*P* = .0018) (Figure 2A‐C).

### 3.2 BRAFi impairs glycolysis and oxygen consumption in BRAFi‐sensitive, but not in BRAFi‐resistant, melanoma cells

**Figure 3:**
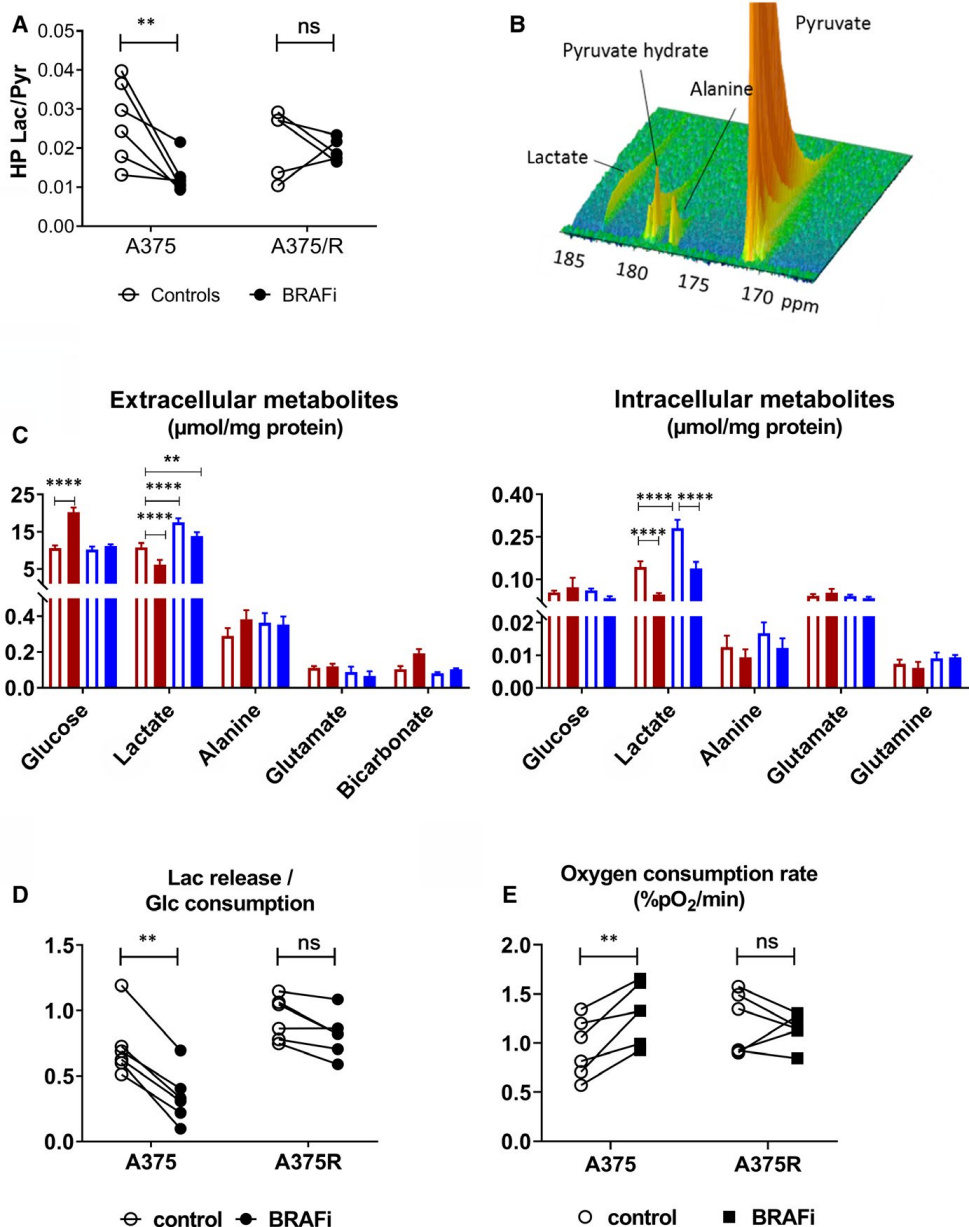
BRAFi impairs glycolysis and stimulates oxygen consumption in sensitive melanoma cells. A, ^13^C label exchange between HP pyruvate and lactate in live melanoma cells pre‐treated with the BRAFi vemurafenib (2 µmol/L, 24 h) or DMSO (two‐way ANOVA, Sidak multiple comparisons test, **P < .01, ns: non‐significant) (n = 6). B, Representative time course of HP ^13^C signal obtained from live A375 (25 × 10^6^) cells. C, Steady‐state concentration of extracellular (left) and intracellular (right) water‐soluble metabolites (two‐way ANOVA, Sidak multiple comparisons test, **P < .01, ****P < .0001, ns, non‐significant) (n = 6). D, For each sample, the ratio between the steady‐state level of extracellular lactate and consumed glucose was calculated to assess glycolytic efficiency (two‐way ANOVA, Sidak multiple comparisons test, **P < .01, ns: non‐significant) (n = 6). E, Oxygen consumption rate measured in live cells (two‐way ANOVA, Sidak multiple comparisons test, **P < .01, ns, non‐significant) (n = 6). Glc, glucose; Lac, lactate; pyr, pyruvate

BRAF inhibition resulted in a decrease of ^13^C label exchange between HP pyruvate and lactate in A375 in vitro, but not in A375R cells (*P* = .0075 and *P* = .8898, respectively) (Figure 3A,B). ^13^C signal arising from alanine was observed in all but one sample. The label distribution between alanine and lactate was also significantly modified by BRAFi in A375, but not in A375R cells (*P* = .0333 and *P* = .8397) (Figure S1A).

Next, we measured the intracellular and extracellular water‐soluble metabolites in melanoma cells incubated with [U‐^13^C] glucose. We observed a reduction in extracellular lactate in both sensitive and resistant melanoma cells treated with BRAFi, when compared to their untreated counterpart (*P* = .0002 and *P* < .0001, respectively). Extracellular glucose was significantly higher in BRAFi‐treated A375 cells (*P* < .0001), thus suggesting that treatment affected glucose uptake, as later confirmed via qRT‐PCR and Western blot analysis of GLUT1 (Figure 3C).

In regard to the intracellular metabolites, lactate pool was decreased both in A375 and A375R cells upon inhibition of BRAF (*P* < .0001 for both A375 and A375R compared with their own controls). Resistant A375R cells; however, had a significantly higher amount of intracellular lactate at baseline (*P* < .0001) (Figure 3C).

The glycolytic efficiency, calculated as the ratio of extracellular lactate over consumed glucose, decreased following BRAFi in sensitive A375 cells (*P* = .0062), but not in A375R cells (*P* = .4765) (Figure 3D). In sensitive cells, the intracellular alanine/lactate ratio was significantly modified after treatment as well (*P* = .0146 for A375 and *P* = .4765 for A375R) (Figure S1B).

The decrease in glycolysis was accompanied by an increase in the oxygen consumption rate in sensitive cells treated with the BRAFi, as demonstrated by in vitro EPR spectroscopy (Figure 3E).

### 3.3 BRAFi affects the transcription of key glycolytic enzymes and transporters in sensitive melanoma cells

**Figure 4:**
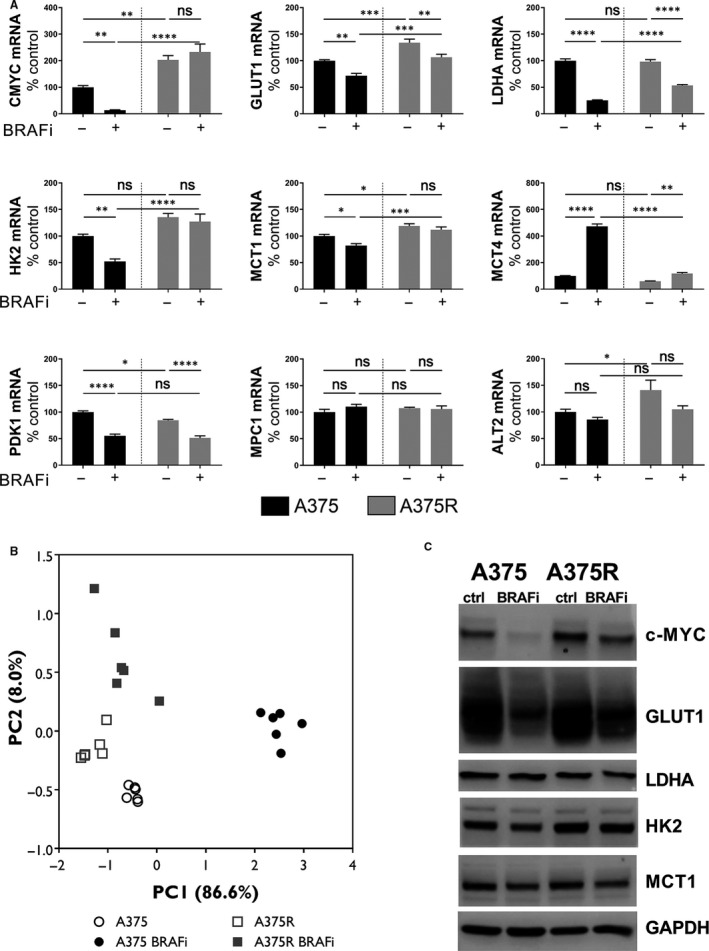
Molecular markers of response to BRAFi in vitro. A, qRT‐PCR analysis of glycolysis‐related genes in A375 (black) and A375R (grey) cells following incubation with BRAFi or DMSO (two‐way ANOVA, Sidak multiple comparisons test, *P < .05, **P < .01, ***P < .001, ****P < .0001, ns, non‐significant) (n = 6). B, Principal component analysis of qRT‐PCR data obtained on A375 cells (control: empty circles, BRAFi: black‐filled circles) and A375 R cells (control: empty squares, BRAFi: grey‐filled squares). C, Western blot analysis of whole cell lysates (right, one representative blot out of three is represented)

Our qRT‐PCR data showed a significant decrease in the mRNA levels of HK2, c‐MYC and MCT1 in BRAFi‐treated A375 cells, compared with untreated controls (*P* = .0046, *P* = .0279 and *P* = .0076, respectively), but neither of them was affected by the treatment in resistant A375R cells. ALT2 and MPC1 mRNA levels were not modified by treatment neither in A375 nor in A375R cells. However, ALT2 levels were higher at baseline in resistant cells (*P* = .0490) (Figure 4A).

Regarding MCT4, LDHA, GLUT1 and PDK1, BRAF inhibition had similar effects on sensitive and resistant cells, even though such effects were of smaller entity in resistant cells, compared with the parental ones. (MCT4: *P* < .0001 for A375 and *P* = .0066 for A375R; LDHA: *P* < .0001 for both A375 and A375R; GLUT1: *P* = .0029 for A375 and *P* = .0064 for A375R; PDK1: *P* < .0001 for both A375 and A375R) (Figure 4A).

Principal component analysis of qRT‐PCR data showed that BRAFi‐treated A375 cells were strongly discriminated by A375R (both treated and control) and untreated A375 by one pattern of mRNA levels (PC1, accounting for 86.6% of the total variation); whereas the second principal component (PC2, accounting for 8.03% of the total variation) helped to discriminate among the other populations (untreated A375, untreated A375R, treated A375R) (Figure 4B).

Western blot analysis of whole cell lysates confirmed the observed reduction in c‐MYC, GLUT1 and HK2 in sensitive melanoma cells (Figure 4C). Despite the reduction at the mRNA level observed for LDHA, HK2 and MCT1, their protein levels were not significantly modified by BRAFi. This could be due to the long half‐life of many glycolytic enzymes, when compared with the much shorter half‐life of c‐MYC.^31^, ^32^

### 3.4 BRAFi reduces hypoxia in vivo, as assessed by electron paramagnetic resonance (EPR) oximetry

Our in vitro results confirmed that BRAFi reduced the glycolytic activity of sensitive melanoma cells, thus resulting in a decrease ^13^C label exchange between pyruvate and lactate. Therefore, we ought to understand whether the increased label exchange observed in vivo was due to treatment‐induced hypoxia.

**Figure 5:**
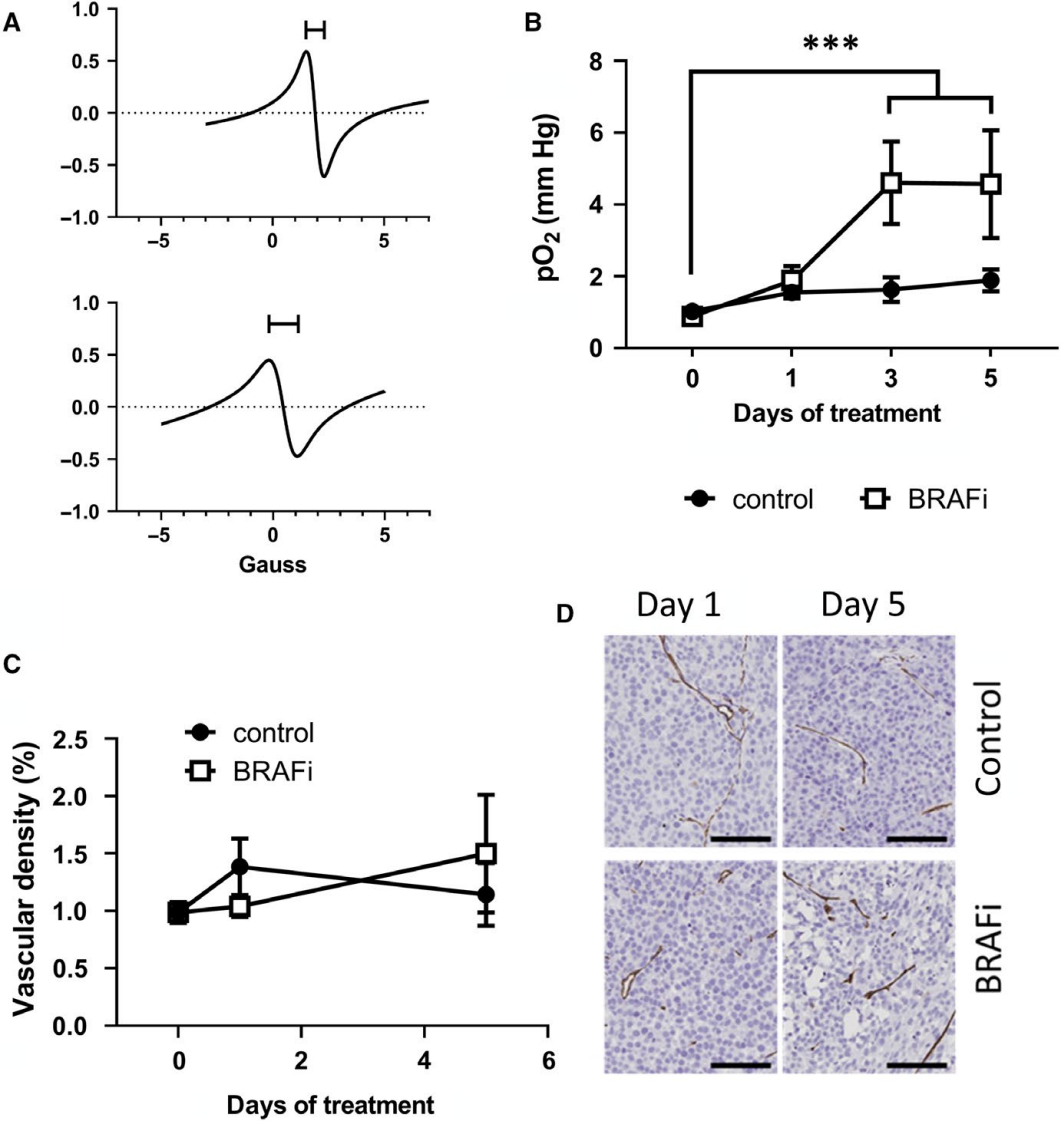
BRAFi reduced hypoxia in vivo, without affecting the vessel density. A, Representative EPR spectra obtained from a mouse prior treatment (top) and after 5 d of treatment with vemurafenib (bottom). Capped lines identify the peak‐to‐peak EPR signal linewidth B, Longitudinal measurements of pO_2_ in control (in blue) and daily BRAFi‐treated (in red) mice (two‐way ANOVA, Sidak multiple comparisons test, ***P < .001) (n = 7). C, Vascular density measured as the ratio of the CD31 positive area/total tissue area, in melanoma xenografts collected at baseline or after 1 d or 5 d of treatment with BRAFi (n = 3‐6) or DMSO (n = 4‐6). D, Representative CD31 staining of melanoma xenografts after 1 or 5 d of treatment. Scale bar: 100 µm

EPR oximetry indicated that melanoma xenografts were highly hypoxic (pO_2_ < 1.5 mm Hg). BRAFi led to an increase in the oxygen partial pressure (pO_2_) of treated tumours, as measured by the peak‐to‐peak signal linewidth of the oxygen sensor (Figure 5A). Such effect occurred after 3 days of treatment, and it was still present after 5 days of treatment (*P* = .0002 at day 3 and at day 5) (Figure 5B).

Immunohistochemical analysis of tumour xenografts collected at baseline and after 1 or 5 days of treatment did not show any significant variation in the angiogenesis marker CD31 (Figure 5D,E). These results suggest that the increased pO_2_ observed via in vivo EPR experiments may be due to a reduced oxygen consumption in vivo, contrarily to what was observed in vitro. A reduction in the cell density caused by BRAFi could have caused a decrease in oxygen demand and hence, an increase in tumour pO_2_. Another possible explanation is that BRAFi has improved oxygenation without affecting blood vessel density.

## 4 DISCUSSION

Inhibition of BRAF led to a significant increase in the HP pyruvate‐to‐lactate label exchange as soon as 24 hours after treatment administration, before any significant tumour shrinkage.

This effect was paradoxical, as BRAFi is known to inhibit glycolysis in BRAF‐mutated melanomas.^17^, ^18^, ^33^, ^34^ Accordingly, our ex vivo analysis of sensitive melanoma xenografts showed significantly lower mRNA levels of GLUT1, HK2 and PDK1 in the treated group, as well as lower C‐MYC and GLUT1 protein levels. Of note, we did not observe any significant effect of BRAFi on LDHA and MCT1/4, the most important factors that influence the ^13^C label exchange between hyperpolarized pyruvate and lactate.^17^, ^35^, ^36^, ^37^

To explain the paradoxical increase in HP pyruvate‐to‐lactate conversion observed in vivo, we assessed hyperpolarized pyruvate in melanoma cells in vitro, thereby excluding the contribution of tumour microenvironment. Contrarily to what was observed in vivo, BRAFi induced a decrease in HP pyruvate‐to‐lactate conversion by sensitive melanoma cells. The impairment of glycolysis was accompanied by an increase in oxygen consumption rate, consistent with BRAFi‐mediated stimulation of mitochondrial metabolism.^16^ Our in vitro hyperpolarization results confirm and extend the work of Beloueche‐Babari and colleagues: they observed a BRAFi‐mediated drop in the HP pyruvate‐to‐lactate exchange in the BRAF‐mutated cell line WM266.4, but not in cells harbouring wild‐type BRAF, therefore, intrinsically resistant to BRAFi.^17^ By using a BRAFi‐resistant clone derived from the parental cell line,^30^ we have shown that the effect in term of reduced glycolysis and label exchange is specific to sensitive cells and may not be present in BRAF‐mutated cells with acquired resistance due to previous exposure to the drug. In line with a reduction of glycolytic activity, following BRAFi in vitro we observed a reduction in mRNA and protein levels of glycolytic enzymes, as well as a decrease in cell glycolytic efficiency, as measured by steady‐state levels of polar metabolites.

We next asked whether the increase pyruvate‐to‐lactate conversion observed in vivo originated from hypoxia. Therefore, we performed immunohistochemical analysis of CD31 and in vivo EPR oximetry, to evaluate changes in tumour vasculature and pO_2_, respectively. We did not observe any significant change in tumour vasculature, nor in tumour pO_2_ 24 hours after treatment, thereby suggesting that the increased pyruvate‐to‐lactate ^13^C label exchange was not caused by a reduced oxygenation. On the contrary, pO_2_ increased at later time‐points, in line with a previous study showing a BRAFi‐mediated relief of tumour hypoxia.^38^ Of note, in our study, the relief of tumour hypoxia was not associated with increased angiogenesis. Moreover, we showed that melanoma cells consume more oxygen after inhibition of BRAF, thus suggesting that the effect was not due to reduced oxygen consumption by melanoma cells either. Several other factors may presumably have modified the in vivo pO_2_: vasodilation, reduced oxygen consumption by stromal cells and reduced number of melanoma cells among other. Besides changes in pO_2_, an increase in the triose compartment of glycolysis in vivo could also have enhanced the conversion of pyruvate into lactate. We have measured only selected enzymes or transporters that are known to be the main factors influencing the fate of hyperpolarized [1‐^13^C] pyruvate, but it would be interesting to evaluate other enzymes in the second phase of glycolysis.

Further studies are needed (a) to identify the factor, or combination of factors, that reduced hypoxia in vivo and (b) to explain the discrepancy in terms of pyruvate‐to‐lactate label exchange between the in vitro and in vivo settings. Regarding the latter, undoubtedly tumour stroma plays a central role in the metabolism of HP pyruvate. It is known that tumours may cope with extremely high lactate levels. As cancer cells do, also fibroblasts and immune cells produce and release lactate in the tumour microenvironment. Metabolic interactions between oxidative/oxygenated and hypoxic/glycolytic tumour cells or between tumour cells and surrounding stroma have been described in several cancer types; however, they have not completely been investigated in melanoma.^39^, ^40^, ^41^, ^42^, ^43^ Interestingly, it has been shown that inhibition of the MAPK cascade in melanoma leads to increased macrophage infiltration within the tumour, both in patients and in mouse xenografts^44^ and both macrophage infiltration and activation can influence the conversion of HP pyruvate into lactate in the myocardium.^45^ Future studies are needed to elucidate the eventual role played by infiltrating macrophages or other stromal cells in the metabolism of hyperpolarized pyruvate in melanoma xenografts.

Within the scope, immunocompetent mice would allow to obtain a more thorough picture of the cross‐talk between cancer cells and immune cells. This is all the more important in immunogenic tumours such as melanoma.

Our study points out some aspects that may be taken into account in future research.

The opposite effect of BRAFi in melanoma cells versus xenografts herein observed corroborates recent literature highlighting the importance of animal studies, with the twofold objective of better understanding the links between oncogenic signals and metabolism, while taking into account tumour heterogeneity, and fostering the clinical translation of newly developed hyperpolarized probes.^29^, ^41^

Finally, discrepancies between the tumour metabolism of HP probes and steady‐state levels of the same metabolites are possible. In fact, contrarily to what happens in physiological conditions, in hyperpolarization experiments the cell metabolism is challenged via the injection of a supra‐physiological dose of the hyperpolarized probe.^46^ The conversion of a hyperpolarization probe and the measurements of steady‐state metabolite levels therefore provide different and complementary information. In our in vitro study, the two approaches provided strikingly similar results, thus suggesting that in this particular model the label exchange between hyperpolarized pyruvate and lactate faithfully described the physiological pyruvate‐to‐lactate conversion.

## 5 CONCLUSION

Following BRAFi, the HP pyruvate‐to‐lactate conversion was significantly increased in human melanoma xenografts. This effect preceded both changes in tumour volume and in tumour oxygenation, thus indicating that the HP lactate/pyruvate ratio may serve as an early marker of response to BRAFi in melanoma. Contrarily to the in vivo settings, BRAFi significantly decreased the HP lactate/pyruvate ratio in vitro, suggesting that such conversion is highly influenced by tumour microenvironment. Future studies are needed to elucidate the precise contribution of stromal cells on the metabolism of HP pyruvate in vivo.

## CONFLICT OF INTEREST

There is no conflict of interest to declare.

## AUTHORS CONTRIBUTION

SA performed the experiments, analysed the data and drafted the manuscript; LM and EL performed experiments; CS and NJ assisted with MRS experiments; FG assisted with qRT‐PCR and WB experiments; CB, JFB, and BG made critical revision to manuscript; BFJ supervised the study, contributed to study design and critical revision. All the authors approved the manuscript for submission.

## Data Availability

The data that support the findings of this study are available from the corresponding author upon request.
