# Supplementary material for: Metabolic imaging using hyperpolarized 13C‐pyruvate to assess sensitivity to the B‐Raf inhibitor vemurafenib in melanoma cells and xenografts
Source: J Cell Mol Med. 2019 Dec 13;24(2):1934–44. doi: 10.1111/jcmm.14890 (PMC6991684; doi:10.1111/jcmm.14890)
Supplement: Supplementary file 1 [file JCMM-24-1934-s001.docx]

## Supplementary materials

| **GENE** | **Sequence (5'->3')** | |
| --- | --- | --- |
| **GLUT 1** | Forward | GGCATGATTGGCTCCTTCTCT |
|  | Reverse | CACGAAGGCCAGCAGGTT |
| **LDHA** | Forward | TATAATCTTCTAAAGGAAGAACAGACC |
|  | Reverse | CCATGCCAACAGCACCAA |
| **HK2** | Forward | CCAGAAGGTGGAGATGGAGA |
|  | Reverse | GGAGCTTCTTGTCTTTGATTTG |
| **c-MYC** | Forward | GCTGCTTAGACGCTGGATTT |
|  | Reverse | CGAGGTCATAGTTCCTGTTGG |
| **PDK1** | Forward | TCTCAGGACACCATCCGTTC |
|  | Reverse | CATCCTCAGCACTTTTGTCC |
| **MPC1** | Forward | AGATTATCAGTGGGCGGATG |
|  | Reverse | GCTGGGCTACTTCATTTGTTG |
| **MCT1** | Forward | CATTTCCATCGGCTTCTCTT |
|  | Reverse | CAAGCAGCCACCAACAATC |
| **MCT4** | Forward | CTTGCTCCTTTAGCCACCAC |
|  | Reverse | GAAACTGGCAAGTCCCAAAA |
| **ALT2** | Forward | TCAAAAAGCCATTCACAGAGG |
|  | Reverse | TGTCCAGCAGGTTTGGGTAG |
| **GAPDH** | Forward | CTGGCGTCTTCACCACCAT |
|  | Reverse | GCCTGCTTCACCACCTTCT |

**Supplementary Table 1** PCR primer design was performed by using the Ensembl database (Release 97) and Primer3 (v.0.4.0). Specificity was verified in Primer-BLAST [49]. Prior use, all primers were tested for efficiency and melt curve quality.

**
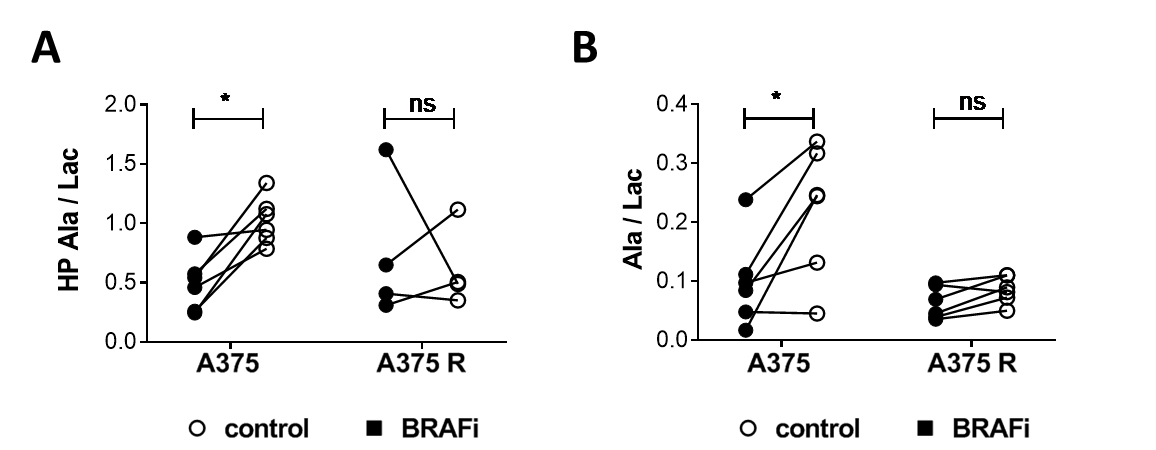
Supplementary Figure 1 Alanine/lactate ratio measured in melanoma cells. A)** the ^13^C label partition between alanine and lactate is significantly modified by BRAFi in A375, but not in A375R, cells (n=6). **B)** Conventional ^13^C NMR data from A375 cells incubated with [U-^13^C]glucose are consistent with hyperpolarization experiments: BRAFi induced an increase in the intracellular alanine fraction compared to lactate only in sensitive A375 cells (two-way ANOVA, Sidak multiple comparisons test, *p<0.05, ns: non-significant) (n=6).Ala: alanine, lac: lactate.
